# Towards better monitoring of technology critical elements in Europe: Coupling of natural and anthropogenic cycles

**DOI:** 10.1016/j.scitotenv.2017.09.117

**Published:** 2018-02-01

**Authors:** Philip Nuss, Gian Andrea Blengini

**Affiliations:** a European Commission, Joint Research Centre (JRC), Directorate D - Sustainable Resources, Ispra 21027, Italy; b Politecnico di Torino, Corso Duca degli Abruzzi 24, 10129 Torino, Italy

**Keywords:** Elemental cycles, Anthropogenic cycles, Natural cycles, Material flow analysis, EU raw materials policy, EU raw materials information systems

## Abstract

The characterization of elemental cycles has a rich history in biogeochemistry. Well known examples include the global carbon cycle, or the cycles of the ‘grand nutrients’ nitrogen, phosphorus, and sulfur. More recently, efforts have increased to better understand the natural cycling of technology critical elements (TCEs), i.e. elements with a high supply risk and economic importance in the EU. On the other hand, tools such as material-flow analysis (MFA) can help to understand how substances and goods are transported and accumulated in man-made technological systems (‘anthroposphere’). However, to date both biogeochemical cycles and MFA studies suffer from narrow system boundaries, failing to fully illustrate relative anthropogenic and natural flow magnitude and the degree to which human activity has perturbed the natural cycling of elements. We discuss important interconnections between natural and anthropogenic cycles and relevant EU raw material dossiers. Increased integration of both cycles could help to better capture the transport and fate of elements in nature including their environmental/human health impacts, highlight potential future material stocks in the anthroposphere (in-use stocks) and in nature (e.g., in soils, tailings, or mining wastes), and estimate anticipated emissions of TCEs to nature in the future (based on dynamic stock modeling). A preliminary assessment of natural versus anthropogenic element fluxes indicates that anthropogenic fluxes induced by the EU-28 of palladium, platinum, and antimony (as a result of materials uses) might be greater than the respective global natural fluxes. Increased combination of MFA and natural cycle data at EU level could help to derive more complete material cycles and initiate a discussion between the research communities of biogeochemists and material flow analysts to more holistically address the issues of sustainable resource management.

## Highlights

• EU raw material policies for enhanced monitoring of technology critical elements are discussed.
• Methodologies to define “technology critical elements (TCEs)” are described.
• Interconnections of anthropogenic and natural elemental cycles are highlighted.
• EU-28 anthropogenic element fluxes for TCEs are compared to global natural fluxes.

## Graphical abstract

**Figure:**
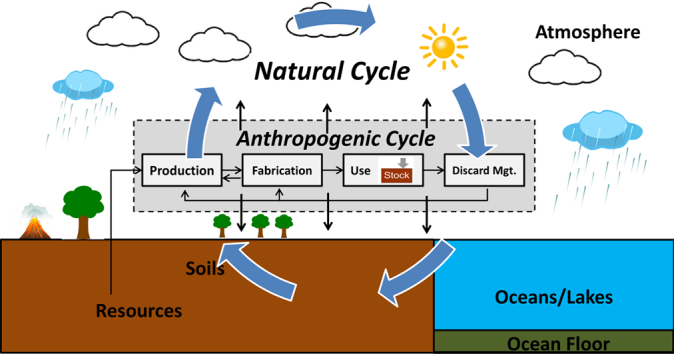

## 1 Introduction

Global population growth, wealthier lifestyles, technological change, and government policies have altered raw materials supply and demand patterns since the early twentieth century (Krausmann et al., 2009, Vidal-Legaz et al., 2016). In particular, the use of multiple materials in single applications to increase product functionality and the push towards low‑carbon technologies and resource efficiency have increased the demand for many of the technology critical elements (TCEs) that did not find widespread use just a few years ago (Greenfield and Graedel, 2013). Global trade networks of goods, in which materials move along the value chain of mining, processing, manufacture, use, disposal, collection, and waste management, have increased in complexity in recent years as multiple countries are involved in the life-cycles of products (De Benedictis and Tajoli, 2011, Fagiolo et al., 2009, Nemeth and Smith, 1985). Furthermore, future economic development and economic growth are expected to take place in areas such as renewable energy technologies, infrastructure for fuel efficient and clean energy vehicles, and low carbon public transportation (UNEP, 2011a), all of which heavily rely on metals (UNEP, 2013).

**Fig. 1:**
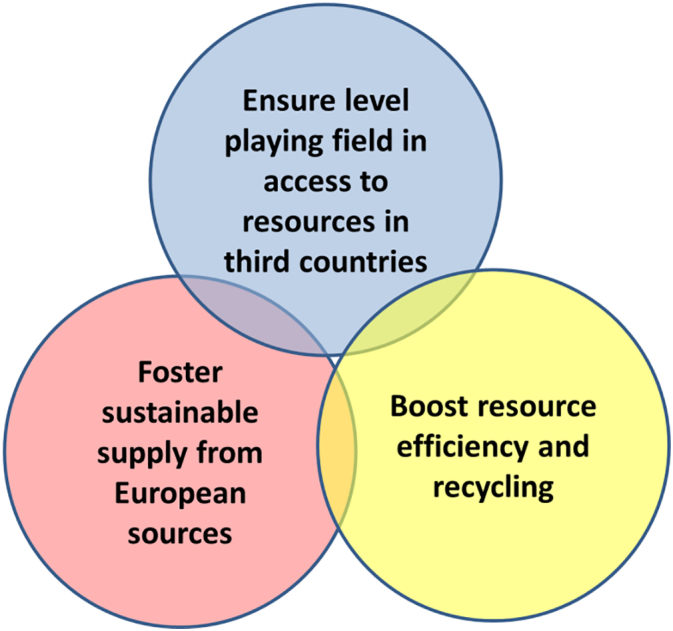
The three pillars of the EU Raw Materials Initiative (RMI).

Against this context, the European Commission (EC) has launched a number of raw materials policies, of which two cornerstones are the EU Raw Materials Initiative (RMI) (EC, 2008) and the Circular Economy (CE) Action Plan (EC, 2015). The RMI aims at securing a sustainable supply of raw materials for Europe and was launched in 2008, and consolidated in 2011. It focuses on non-energy and non-agricultural materials and connects to EU external and internal policies, e.g., related to raw materials trade (EC, 2014a) and EU structural and investment funds (EC, 2014b). The RMI is an integrated strategy consisting of 3 pillars, which target sustainable supply of raw materials from outside and inside Europe, and aims to boost resource efficiency and recycling (Fig. 1). The initiative introduced and updated the list of critical raw materials (CRMs) for the EU in 2011, 2014, and 2017 (forthcoming) which identify materials characterized by a high supply risk and a high economic importance for Europe (Blengini et al., 2017c, EC, 2010, EC, 2014c).

More recently, the CE action plan aims to stimulate Europe's transition towards a more circular economy (EC, 2015). Circular economy is defined as a state in which “the value of products, materials, and resources is maintained in the economy for as long as possible, and the generation of waste is minimized” (EC, 2015). The CE action plan is currently being implemented. In 2017, the Commission plans to present a strategy for plastics, report on CRMs in the circular economy, an assessment of options for the improved interface between chemicals, products and waste legislation, a legislative proposal on water reuse, and a monitoring framework on circular economy (EC, 2017).

EU policies such as the RMI and CE action plan rely on information and data on material flows and stocks within the EU economy and their level of circularity within Europe (*anthropogenic cycles of the elements*) (EC, 2012). Material flow analysis (MFA) approaches (Brunner and Rechberger, 2004, Brunner and Rechberger, 2016, Müller et al., 2014) have been used widely over the past decade to characterize the anthropogenic life cycles of both substances and goods (Chen and Graedel, 2012). MFA aims at examining the anthropogenic stocks and flows of materials at each stage of their life cycle in order to gain a more complete understanding of their status above ground. Material flows and stocks can be illustrated using Sankey diagrams (Schmidt, 2008) if the number of transformation processes is small, or network visualizations (Nuss et al., 2016) for datasets involving a larger number of transformations steps and material flows between them. Understanding the whole system of material flows can help to quantify potential primary and secondary source strengths, manage metal use more wisely, and protect the environment (Brunner and Rechberger, 2004, Brunner and Rechberger, 2016). The EC has recently published MFAs for the EU-28 (28 member states which comprise the European Union (EU)) for 28 materials (referred to as Material System Analysis (MSA) studies in the report by (BIO by Deloitte, 2015)) and a EU MFA data platform is currently in development in the Raw Materials Information System hosted at the EC Joint Research Centre (Manfredi et al., 2017).

The anthropogenic cycles of elements are embedded into their larger natural biogeochemical cycles (*natural cycles of the elements)* (Schlesinger, 2005) which describe, e.g., the exchanges of the anthroposphere (often also referred to as the “technosphere”) with the environment and subsequent transport, fate, and accumulation of elements in nature. Natural cycles include natural element flows, e.g., due to riverine flux to oceans, eolian dust, seaspray, net primary productivity (NPP), extraterrestrial matter, volcanoes, and soil erosion (Sen and Peucker-Ehrenbrink, 2012). The characterization of elemental cycles has a rich history in biogeochemistry. Famous examples include the global carbon cycle, or the cycles of the ‘grand nutrients’ nitrogen, phosphorus, and sulfur. Knowledge of natural cycles has evolved over many decades as new sinks and sources have been discovered and missing flows between reservoirs have been quantified. For example, the current *COST action TD1407 “Network on technology-critical elements (TCEs): From Environmental Processes to Human Health Threats”* funded under the EU Framework Programme Horizon 2020 attempts to expand the knowledge-base on the natural cycling of technology-critical elements (TCEs) (i.e., platinum group elements (PGEs), rare earth elements (REEs), Nb, Ta, Ga, Ge, In, Tl, Te) (Cobelo-García et al., 2015). It also aims to create a network of scientists working and interested on TCEs with the aim of defining the current state of knowledge and gaps, proposing priority research lines/activities, and acting as a platform for new collaborations and joint research projects.

However, to date both biogeochemical cycles and MFA studies suffer from narrow system boundaries, failing to fully illustrate the interlinkages between natural and anthropogenic cycles, relative anthropogenic and natural flow magnitude (Rauch and Graedel, 2007, Rauch and Pacyna, 2009, White and Hemond, 2012), and the degree to which human activity has perturbed the natural cycling of elements (Klee and Graedel, 2004, Rauch and Pacyna, 2009, Sen and Peucker-Ehrenbrink, 2012). For example, while MFA studies indicate the amounts of an element released from the anthroposphere into the environment (flows crossing the system boundary), the subsequent transport and fate, and possible accumulation in the environment are often poorly studied. Given that increasing amounts of TCEs are today transported and accumulated in man-made technological systems, knowledge about anticipated future exchanges is also of importance accounting for material end-use patterns in modern technologies and related in-use stock dynamics. Furthermore, considering only part of an element's life-cycle (narrow system boundary) increases the risk for burden shifting. Examples are manifold and include, for instance, burden shifting between different environmental impact categories (using chemically cleaned coals might reduce air emissions of toxic metals but at the expense of increased energy requirements and climate impacts), between different media (SO_2_ scrubbers create solid waste), between life-cycle stages (embedded electronics improve operating efficiency but create waste disposal concerns), or between generations (long-term storage of toxic waste). In addition, in the absence of detailed toxicological studies, a comparison of anthropogenic fluxes and concentrations to natural values can also help to understand the probability of adverse toxicological effects of elements (Klee and Graedel, 2004, Nriagu, 1996, Pacyna and Pacyna, 2001, White and Hemond, 2012). Therefore, a better understanding of both natural and anthropogenic cycles including their stocks and interlinkages can help to more holistically view element cycles and connect the research communities of both biogeochemists and MFA practitioners/resource managers with each other.

Against this background, this paper attempts to highlight some of the important interconnections between natural and anthropogenic cycles and related data sources at EU level. It highlights a number of relevant raw material dossiers by the EC that aim at increasing the knowledge base on raw materials (e.g., Material System Analysis (BIO by Deloitte, 2015), the Raw Materials Information System (RMIS) (Manfredi et al., 2017), and EU criticality assessments (Blengini et al., 2017c, EC, 2011, EC, 2014d)). Given that MFA studies on several TCEs are already available (from the MSA), further MFA studies currently being carried out at EU level by the EC, and natural cycle counterparts being generated in the COST action, synergies might develop that could, ultimately, lead to a combination of both data sets to derive more complete material cycles for the EU and globally, thereby initiating a discussion between the research communities of biogeochemists and material flow analysts to more holistically address the issues of sustainable resource management.

For this, we firstly provide a definition of TCEs based on resource criticality assessments. Criticality assessments provide a first investigation of the importance of a material in today's uses (e.g., by a company, country, region, or globally), highlight potential issues if the material were not available, and describe issues of supply risk. Secondly, interlinkages of natural and anthropogenic cycles are highlighted and we discuss how both provide complementary sets of information, e.g., to anticipate possible future emissions from the anthroposphere to nature, identify secondary (“hibernating”) resource stocks at the anthroposphere-environment interface (e.g., in mine waste or landfills), and better understand the environmental and human health impacts of metals dissipated to the environment during a material's full life-cycle. Thirdly, we discuss how data collected by biogeochemists and material flow analysts could be increasingly combined to approximate the magnitude of anthropogenic and natural metal mobilization in the EU and estimate human vs. natural dominance in perturbing the cycles of the elements. Finally, we conclude with ideas for future research that would help in strengthening the dialogue between the research communities of biogeochemists and material flow analysts.

## 2 Definition of technology-critical elements (TCEs)

In 2008, the United States National Research Council proposed a framework for evaluating material “criticality” based on the material's supply risk and impact of a supply restriction (NRC, 2008). Since then, a number of organizations worldwide have built upon that framework to evaluate raw materials' criticality in various ways (BGS, 2012, Blengini et al., 2017c, DOD, 2013, EC, 2011, EC, 2014d, Graedel et al., 2015; IW Consult, 2011, Morley and Eatherley, 2008, NSTC, 2016, Skirrow et al., 2013). Common to the majority of criticality assessments is the focus on a material's supply risk and the “importance” of the material, e.g., to a corporation, country, region, or globally.

Supply risk principally relates to concentration of production in a restricted number of countries, their geopolitical and social and regulatory structure, whether a material is produced on its own or is dependent on the demand for another material (host-companion relationships), and the extent to which a material can be substituted in certain end-use applications. Some assessments also consider the life-cycle wide environmental implications (cradle-to-gate) of materials production (Nuss and Eckelman, 2014). On the other hand, the impact of a supply restriction is considered differently in the various assessments (Dewulf et al., 2016) and the EU criticality methodology looks at a material's economic importance to the EU economy as a whole (Blengini et al., 2017a, Blengini et al., 2017b, EC, 2010, EC, 2014c).

**Fig. 2:**
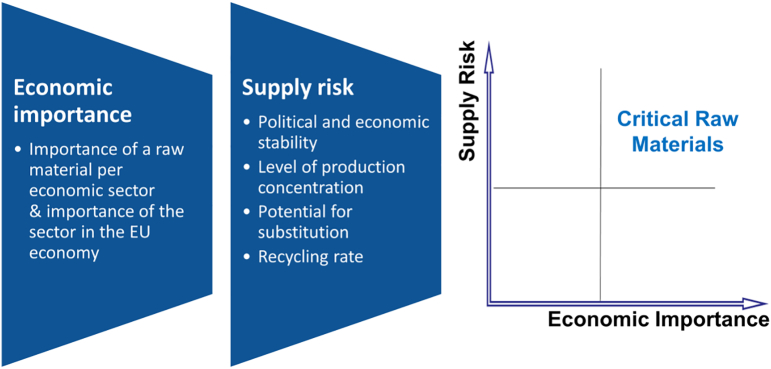
Criticality assessments define “technology critical elements (TCEs)”.

The EC evaluates the criticality of various raw materials to the EU economy every three years (EC, 2011, EC, 2014c). The list of critical raw materials (CRMs) (referred to as TCEs by the COST action) for the EU (EC, 2011, EC, 2014c) and the underlying criticality methodology (Blengini et al., 2017a, Blengini et al., 2017c, Chapman et al., 2013, EC, 2010) are key instruments in the context of the EU raw materials policy. Such a list is a precise commitment of the Raw Material Initiative (RMI) (EC, 2008) and subsequent updates. A list of typical parameters taken into account in the EU criticality methodology (an economy-wide assessment) is shown in Fig. 2. Other criticality methodologies and frameworks exist, e.g., looking at single corporation (Duclos et al., 2010), a sector or a few selected technologies of strategic importance (sector-specific criticality assessment) (Moss et al., 2013a, Moss et al., 2013b, USDOE, 2010, USDOE, 2011), to entire national/regional economies (economy-wide criticality assessment) (Achzet et al., 2011, BGS, 2012, Coulomb et al., 2015, EC, 2010, EC, 2014c, Graedel et al., 2015, NRC, 2008, NSTC, 2016, Skirrow et al., 2013), and the world (global criticality assessment) (Graedel et al., 2015).

Obviously, as supply and demand change over time (e.g., mines opening/closing or new technologies entering the market) the EU list of critical raw materials (CRMs) can change with each assessment (Table 1). Such a change could be the result, e.g., of a new technological breakthrough (resulting in sudden increases in demand), shifting production patterns (e.g., fewer countries producing a material, or shifts to producing countries with high governance risks), no proper material substitutes being available, and problems with the recovery and recyclability of the material at end-of-life. For example, Ta was considered a critical raw material in 2011 but is not part of the updated list in 2014. This is a result of changes in the shares of global primary production of Ta. In the first criticality exercise D.R. Congo (with poor governance rating) has been a major Ta producer, while in the second assessment in 2014 Brazil and Australia (with better governance ratings) are also significant suppliers thereby reducing supply risk. More information on each raw material are also provided in the annexes of the respective criticality lists (EC, 2011, EC, 2014c).

**Table 1:** Lists of critical raw materials (CRMs) according to the EC methodology.

EC criticality assessment	(EC, 2011)	(EC, 2014c)	2017 assessment (forthcoming)
Critical raw materials (CRMs) identified	Sb, Be, Co, Fluorspar, Ga, Ge, Natural graphite, In, Mg, Nb, PGMs^a^, REEs^b^, Ta, W	Sb, Be, B, Cr, Co, Coking coal, Fluorspar, Ga, Ge, In, Magnesite, Mg, Natural graphite, Nb, P, PGMs^a^, REEs^b^, Si, W	Methodology described in (Blengini et al., 2017a, Blengini et al., 2017b, Blengini et al., 2017c). 2017 list to be published

a Platinum group elements (Pt, Pd, Rh, Os, Ir, Ru).

b Rare earth elements according to the EU CRM assessment (Y, La, Ce, Pr, Nd, Sm, Eu, Gd, Tb, Dy, Yb, Lu, Ho, Er, Tm).

The EU CRM assessment helps to highlight the relative “importance” of materials for which more detailed information, e.g., on their anthropogenic and natural cycles would be beneficial for more sustainable resource management and to alleviate potential supply risks to the EU. The assessment is backward looking and could be combined with more prospective assessments to highlight materials with possible criticality in the future to inform the scientific community of material flow analysts and biogeochemists on the material cycles of immediate and future interests.

## 3 Interlinkages of natural and anthropogenic element cycles

Proper monitoring of anthropogenic as well as natural material stocks and flows in Europe and globally is crucial to foster resource efficiency and a circular economy, reduce environmental pressures arising throughout a material's life-cycle, and quantify the availability, or “elemental criticality,” to the EU economy. However, to date important information from both biogeochemical cycles and MFA studies about relative anthropogenic and natural flow magnitudes, sources and sinks of critical elements in Europe, dissipation and accumulation in both the environment and anthroposphere, and the degree to which human activity has perturbed the natural cycling of elements are missing. To date, only a handful of global elemental cycles, inclusive of natural and anthropogenic stocks and flows, have been constructed quantitatively (Rauch and Graedel, 2007, Rauch and Pacyna, 2009, White and Hemond, 2012), but no cycles of this kind exist at the level of the EU.

### 3.1 EC Raw Material System Analysis (MSA)

**Fig. 3:**
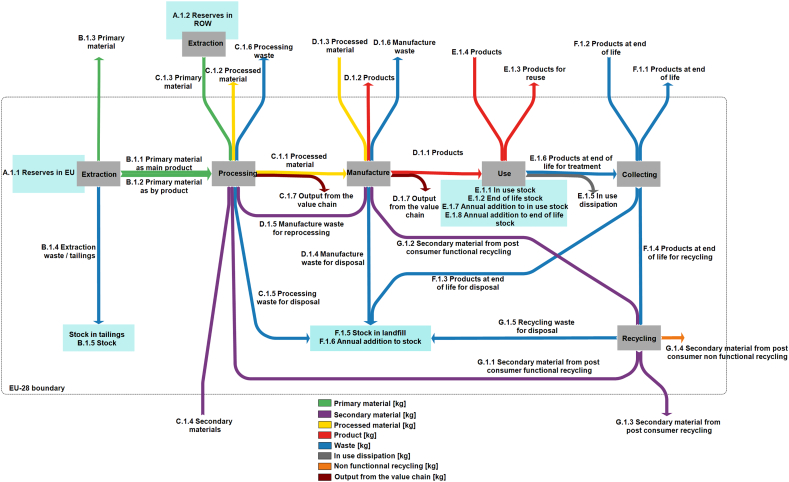
Schematic figure of the MSA framework and flows/stocks considered.

Through the MSA, the EC has recently amplified efforts to assemble data on anthropogenic material stocks and flows in the EU economy to more effectively manage its resource base (BIO by Deloitte, 2015, EC, 2012). The MSA was carried out in 2015 and investigates the flows and stocks of 28 raw materials from “cradle-to-grave”, that is, across the entire material life cycle from resource extraction to materials processing to manufacturing and fabrication to use and then to collection, processing, and disposal/recycling (Fig. 3).

(Source: (BIO by Deloitte, 2015.)

It is a follow-up of the “Study on Data Needs for a Full Raw Materials Flow Analysis”, launched by the European Commission in 2012 within the context of the EU RMI strategy (EC, 2012). The objective of the MSA study is to provide information on material stocks and flows and to assist the EC on the development of a full MSA for a selection of key raw materials used in the EU-28. Anthropogenic stocks and flows information is particularly important for CRMs for which reliable information on their trade is sometimes incomplete or unavailable, their uses are not well understood, and their recovery and reuse once discarded is problematic. The EU MSA study includes reserve estimates (that part of an identified resource that meets specified minimum physical and chemical criteria related to current mining and production practices (USGS, 2012a)) but does not include reserve base or resource numbers.

An accurate assessment of global and EU-wide mineral resources must include not only the resources available in ground (ore reserves and mineral resources) (Mudd et al., 2017a, UNECE, 2017, USGS, 2012b), but also those that are present as stocks within the technosphere and might become available through recycling (Fishman et al., 2014, Krausmann et al., 2017, Maung et al., 2017, Rauch, 2009, UNEP, 2010). According to Ayres et al., there are four categories of anthropogenic metal stocks, namely long-lived goods in use (e.g., buildings and infrastructure), short-lived goods in use (e.g., certain consumer electronics), landfills and identifiable mine waste dumps, and metals that have been irrecoverably lost by dissipation into the environment (Ayres et al., 2002). In some cases, materials dissipated into the environment might be recovered as technologies advance (e.g., recovery of platinum group elements such as platinum and palladium from roadside dust (Hunt, 2013)), while in other cases recovery might not be possible due to the high energy requirements involved (Capilla and Delgado, 2014). Different MFA modeling approaches exist to quantify stock accumulations and flows within the technosphere (Müller et al., 2014). The data resulting from the MSA study for CRMs (BIO by Deloitte, 2015) provides an important base of background information from which future materials criticality can be better addressed, and sustainable development pathways, on an EU-wide scope, designed.

### 3.2 Biogeochemical cycles in the EU

Simultaneously, the current *COST action TD1407* funded under H2020 attempts to expand the knowledge-base on the natural cycling ofTCEs and create a network of scientists interested in the natural and anthropogenic cycles of TCEs in Europe (Cobelo-García et al., 2015) (see this special journal issue for more details).

### 3.3 Interlinkages of natural and anthropogenic cycles

**Fig. 4:**
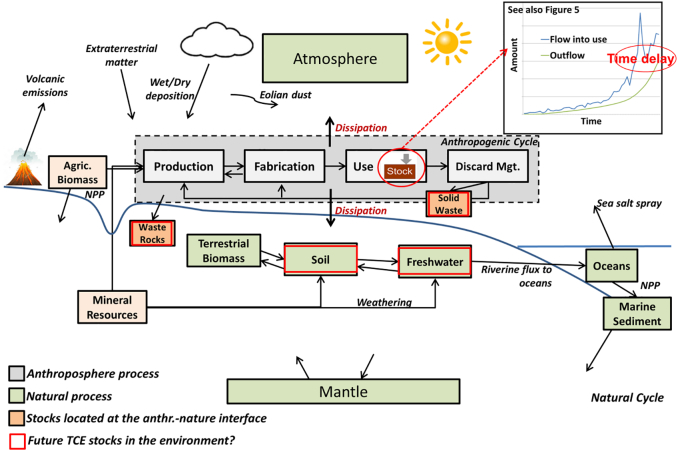
Schematic illustration of the anthropogenic cycle of an element located within the larger natural cycle (inspired by (Rauch and Pacyna, 2009, White and Hemond, 2012)). NPP: Net primary productivity.

A combination of both natural and anthropogenic cycles can help to quantify the link between societal flows and stocks of metals and their natural cycling (van der Voet et al., 2013). This is in particular important to highlight options for how the societal cycle could be further isolated from the natural cycle (given that virgin resource use and releases to nature often result in adverse environmental and social impacts), quantify both natural and anthropogenic stocks of TCEs, and show how the elements continue to cycle after exiting the anthroposphere. Fig. 4 shows a schematic illustration of the anthropogenic cycle embedded into their larger natural cycle.

*Firstly*, the **transport and fate of TCEs** in the natural environment is often poorly studies. MFA models can provide estimates of the amount of an element crossing the system boundary, e.g., in the process of being dissipated into the environment during processing, manufacture, or the use stage, and as losses to air/soil/water during waste management (e.g., metals leaching into soils from a landfill over time) (Fig. 4). However, often these loss values are approximated in the process of mass balancing the MFA model. Instead, natural cycles can provide actual measured values on these losses which can inform the MFA model.

The subsequent fate of an element in the environment is not usually taken into account in MFA studies but is important to quantify downstream impacts on human health and the environment, as well as to identify reservoirs in nature with high element concentrations. For example, natural cycles can identify element flows from the technosphere into freshwater bodies or soils, accumulation in these reservoirs, as well as uptake in local fish or plant species.

Life-cycle assessment (LCA) is a tool for systematically evaluating the potential environmental burdens of products, technologies, and services (Baumann and Tillman, 2004, Curran, 2012, ISO, 2006a, ISO, 2006b) and is sometimes combined with MFA (Laner and Rechberger, 2016, Lopes Silva et al., 2015, Venkatesh et al., 2009). Combinations of MFA with chemical risk indices are less frequent (Eckelman and Graedel, 2007). However, the fate of pollutants in the environment and their impacts are generally site-specific and can therefore be difficult to quantify using generic fate-transport models (Goedkoop et al., 2009, Rosenbaum et al., 2008). There is also a lack of knowledge and data on the toxicological properties of many of the TCEs (Nuss and Eckelman, 2014, van der Voet et al., 2013). Better on-site measurements in combination with models by the biogeochemical community could help to enhance existing estimates especially for the TCEs in Europe. This is relevant to the overall issue of environmental sustainability.

*Secondly*, **natural cycles could highlight metal “stocks”** in nature i.e., environmental compartments with high TCEs levels such as soils, sediments, water bodies, but also mine waste rocks, tailings, or landfills (both those still managed and those that have been abandoned and remain in nature) located at the anthroposphere-nature interface, might become resource deposits from which secondary raw materials can be recovered in the future (indicated with red rectangles in Fig. 4). Geological stocks including reserves and sometimes reserve base are estimated and monitored by geologists (e.g., (BGS, 2014, UNECE, 2017, USGS, 2011), but for TCE which are frequently produced as by-products they are often not available (Mudd et al., 2017a). However, given that industrial applications today rely on a large part of the elements of the periodic table (UNEP, 2011b) and metals losses take place, e.g., as mine wastes, dissipative losses from mining and smelting (van der Voet et al., 2013), and during the use phase (Ciacci et al., 2015), certain environmental compartments such as soils, water bodies, or mine wastes and landfills might become increasingly relevant “suspects” for the recovery of metals, especially TCEs in Europe. In fact, there is increasing interest in better quantifying and systematically classifying anthropogenic stocks including long- and short-lived goods in use, and landfill and identifiable mine waste dumps (Fishman et al., 2014, Krausmann et al., 2017, Maung et al., 2017, Rauch, 2009, UNEP, 2010). However, to date it is unclear whether environmental concentrations of TCEs are in fact increasing due to their use in new technologies (Filella and Rodríguez-Murillo, 2017). The recovery of TCEs from alternative sources such as old tailings or contaminated soils is also often challenging from an engineering and economical viewpoint (Mudd et al., 2017a, Mudd et al., 2017b).

However, information on potential natural stocks of TCEs in Europe forming as the result of continued losses to the environment such as in soils, sediments, or abandoned mine waste dumps, are not yet widely available (Fig. 4). The EU soil atlas provides some data and maps on organic soil material and soil material for construction in Europe (EASDAC, 2016), while the Geochemical Atlas of Europe provides some data on TCE levels in different environmental compartments including soils (EGS, 2016). Recently, the GEMAS (geochemical mapping of agricultural soil) project published concentrations of 53 elements in European agricultural soils (Reimann et al., 2017). The TCE COST Action will deliver data on concentrations of TCEs in the European environment (e.g., soils, water, waste treatment, sediments, and biota). This can show anomalies either from geological or anthropogenic sources, highlight geographical hotspots of metal losses, and impacts of TCE use on soil sustainability. Such data could then be increasingly incorporated into “maps” of the anthropogenic stocks and flows of materials in the EU.

**Fig. 5:**
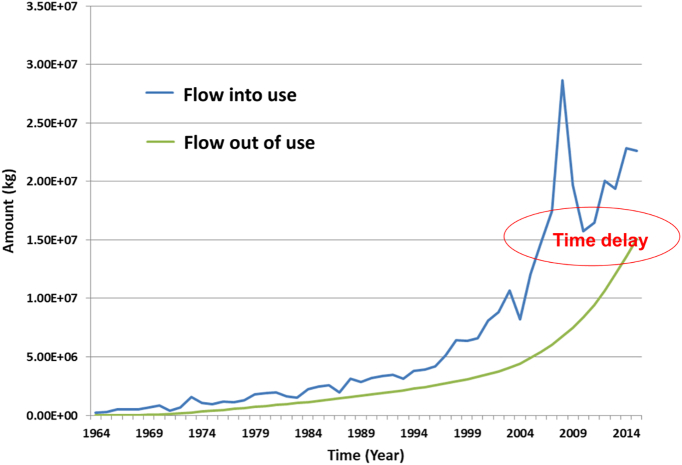
Simple dynamic stock model for niobium showing the global flow into use for transportation purposes and the modeled (anticipated) outflow using an average lifetime of 10 years for vehicles (based on data provided in (Nuss et al., 2014)). Supplementary data are provided in Appendix A.

*Thirdly*, dynamic stock modeling to calculate in-use stocks in **MFA can help to estimate future waste streams and emissions** (see the “use” process in the anthropogenic cycle shown in Fig. 4 and related graph as well as Fig. 5). As increased consumption has led to an accumulation of significant stocks of metals in the anthroposphere (in the form of building, infrastructure, consumer goods, and others), future outputs, when goods reach their end-of-life, are becoming more important. The level of collection and recycling of metals from these secondary resources depends on various factors such as market prices for the materials, product compositions, recycling infrastructures in place (Reck and Graedel, 2012, Reuter et al., 2013), and other issues. However, a significant fraction of societal material inputs (from imports and domestic extraction) are not recycled at the end of product life and become societal outputs (waste and emissions) in the future. Anticipating such future emissions is important for environmental policy to anticipate future problems and take timely action (van der Voet et al., 2002). Future outputs of materials from the use-phase can be quantified by using dynamic MFA models in which the outflows of the in-use stocks are based on inflow or stock data and lifetime distribution functions (Müller et al., 2014).

From the perspective of the biogeochemical community, such information provides possible scenarios for elements that might dissipate into the environment in the future (i.e., crossing the system boundary from anthroposphere to nature) if not managed properly during end-of-life treatment. It can thereby help to prioritize future research efforts to monitor specific single elements in the environment. Fig. 5 shows an example of niobium in transportation (e.g., cars) at global-scale and the related flows into use, and modeled outflows at end-of-life based on a simple dynamic stock model using a lifetime assumption of 10 years (Nuss et al., 2014).

Fig. 5 shows that metal containing goods exit the use-phase with a time delay (depending on the life-time of the goods in use) and outflows continue even after flow into use decreases (e.g., between 2009 and 2010 for niobium in automobiles). This delay depends on the lifetime of the goods in use (e.g., materials in construction might have lifetimes of 50 years or more, while those in typical consumer goods, e.g., electronics, might only have lifetimes of a few years).

In reality, the dynamics of material stocks depend, obviously, on many variables such as technological development (e.g., recycling infrastructure and product design), population size, welfare, market development (prices, demand, costs), government policies, and others (not taken into account in Fig. 5). Nevertheless, the example shows the general idea that niobium becomes available in the future with a small time-delay. Globally, roughly 53% of the outflow is recycled (end-of-life recycling rate) while the remaining 47% become part of the waste stream which may ultimately end-up in landfills. Stocks can be very large and eventually will become waste and emissions that, if not managed properly, can dissipate into the environment where they might continue to cycle. For example, past studies have shown that lead in cathode-ray tubes (CRTs) continues to be released from existing in-use stocks even 1–2 decades after the inflow ended (note that today CRTs have been largely replaced by flat screen displays) (Elshkaki et al., 2005).

## 4 Anthropogenic disturbance of elemental cycles at the Earth's surface

The coupling of natural and human cycles of the TCEs can help to show the **degree to which human activity has perturbed the natural cycling of elements** (Klee and Graedel, 2004, Sen and Peucker-Ehrenbrink, 2012) given current demand for primary resources. Past studies have highlighted the increasing dominance of humans in shaping their environment and relate to the scientific debate on the anthropocene and planetary boundaries (given how slow geological change normally is, at least when measured against an average human lifespan, the suggestion that people are now equivalent to a “natural force”) (Castree, 2016, Hooke, 2000, Nir, 1983, Wilkinson, 2005).

With reference to the EU, it is yet unclear what the EU's contribution to global anthropogenic metal mobilization for the TCEs is and how this compares to global (and EU-wide) natural mobilization estimates. One possibility to capture the metal mobilization induced by the EU-28 is to use estimates on anthropogenic mass transfer values for the 28 materials covered in the MSA study (BIO by Deloitte, 2015) and compare these with global natural mobilization estimates (Sen and Peucker-Ehrenbrink, 2012). The latter include riverine flux to oceans, eolian dust, sea-salt spray, primary productivity, extraterrestrial matter, volcanoes, and soil erosion (base year approximately 2011, given in Gg/yr).

**Fig. 6:**
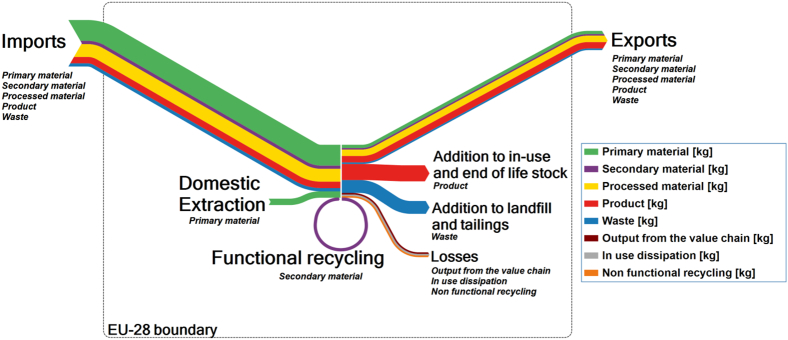
Simplified Sankey diagrams from the MSA study (BIO by Deloitte, 2015) showing the flows (in kg/year, usually in metal content) used in the calculation of anthropogenic mobilization of TCEs by the EU-28.

For anthropogenic mobilization, the EU-28 is consuming not only primary raw materials but relies also on imports of, e.g., processed materials and semi-finished and finished products as well as secondary materials. Furthermore, not all materials entering the EU-28 economy are consumed domestically (within EU borders), but exported to other countries located outside of the EU also take place (Fig. 6).

In this study, we approximate anthropogenic element mobilization by the EU-28 using the following material flows entering and exiting the EU economy (see Fig. 6 and Tables S2 and S3 in Appendix A):

a) The sum of domestic extraction, imports of primary material, imports of processed material, and imports of products (semi-finished and finished). This reflects **direct material input (DMI)** excluding secondary material inputs.
b) The **domestic material consumption (DMC)** calculated as: DMC = Domestic Extraction + Imports – Exports.

This reflects the mobilization of elements as a result of (a) direct material input to the EU of primary materials or (b) domestic consumption of materials (i.e., subtracting material exports which are processed in the EU but consumed elsewhere). However, we note that in this preliminary assessment a number of additional anthropogenic element flows such as biomass burning, coal and petroleum burning, human productivity, and construction are not taken into account. The reason for this is that MFA focuses on the direct material flows associated with the extraction, processing, use, and end-of-life management phase of a particular material, as well as imports and exports for different life-cycle stages, but does not generally account for additional flow associated with the activities mentioned above (e.g., element mass mobilization from rock and sediment displacement during construction activities, burning of fossil fuels, etc.). Such flows have been included in other studies at global level and shown to be significant for anthropogenic mobilization estimates of a number of elements (Klee and Graedel, 2004, Sen and Peucker-Ehrenbrink, 2012). Therefore, this assessment should be seen only as a first conservative estimation of EU anthropogenic mobilization to be further refined in the future. Using data from MFA, however, allows one to tackle the issue of country/regional system boundaries where elemental flows as a result of consumption within a territory need to be properly accounted for.

**Fig. 7:**
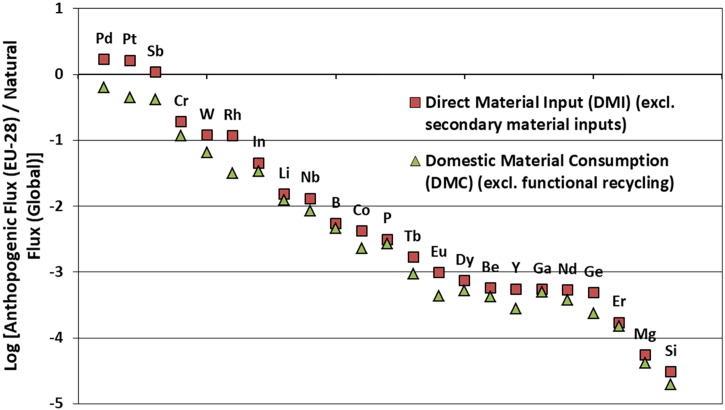
A first comparison of the ratio of EU-28 anthropogenic mobilization fluxes (only due to direct materials use, i.e., not including other factors such as element mass mobilization from coal burning, construction, etc.) to global natural mobilization values. Positive logarithms indicate human fluxes (by the EU-28) that are greater than respective natural fluxes (global). Human mobilization fluxes as a result of EU-28 economic activity are based on MFAs for year 2012 (BIO by Deloitte, 2015). Natural mobilization figures come from (Sen and Peucker-Ehrenbrink, 2012) in approximately 2011 and include riverine flux to ocean, eolian dust, seaspray, net primary productivity (NPP), extraterrestrial matter, volcano, and soil erosion (i.e., does not include, e.g. the Earth's mantle as shown conceptually in Fig. 4). Supplementary data used in the figure and additional calculations are provided in Appendix A.

Our assessment of natural versus anthropogenic element fluxes indicates that anthropogenic fluxes induced by the EU-28 of palladium (Pd), platinum (Pt), and antimony (Sb) might be greater than the respective global natural fluxes (Fig. 7 and Tables S2 and S3 in Appendix A). For these elements, EU requirements for materials purposes alone (because we do not include mobilization due to e.g., coal burning or construction) might already lead to metals mobilization at the scale of current natural fluxes. However, uncertainties in both the MFA calculations (which could not be quantitatively assessed as of yet) and the natural mobilization fluxes do not allow us to definitively stating that human activities within the EU dominate the biogeochemical cycling of these elements. While for Pd, Pt, and Sb (identified on the higher end in this assessment), anthropogenic mobilization due to mining was found to be the major factor, other factors (i.e., biomass burning, coal and petroleum burning, human productivity, and construction) can also be significant (Klee and Graedel, 2004, Sen and Peucker-Ehrenbrink, 2012). Furthermore, losses during material extraction and processing are often not properly accounted for but can be significant (for example, (Sen and Peucker-Ehrenbrink, 2012) provide an approach to allow for corrections of unrecognized material losses during material extraction and processing for the platinum-group metals and rare earth elements). Including these flows might further increase the human mobilization numbers provided here.

On the natural cycling side, global estimates were used as EU specific data does not yet exist. The latter could be increasingly provided for the EU by the current COST action (Cobelo-García et al., 2015). Globally, Sen and Peucker-Ehrenbrink find that surface anthropogenic fluxes of iridium (Ir), osmium (Os), helium (He), gold (Au), ruthenium (Ru), antimony (Sb), platinum (Pt), palladium (Pd), rhenium (Re), rhodium, and chromium (Cr) currently exceed natural fluxes (Sen and Peucker-Ehrenbrink, 2012).

## 5 Discussion and conclusion

Sustainable resource management requires consideration of various aspects related to the environmental, social, and economic aspects of Europe's resource demands. Given that the EU is highly dependent on imports of a large number of materials used in a modern economy, issues of resource criticality are high on the political agenda today. For this, proper knowledge of the natural material flows and stocks as well as the human industrial and societal metabolism provide important background information. A combination of both natural and anthropogenic cycles of the elements can help to provide more complete “maps” of Europe's resource base, e.g., by showing potential future material stocks both in the anthroposphere (in-use stocks) and in nature (e.g., in soils, tailings, or mining wastes). Biogeochemical measurements can help to better capture the transport and fate of elements released into the environment as a result of anthropogenic activities and highlight potential environmental and human health impacts. They also provide an important “reference point” for MFA practitioners who, due to a lack of data, often need to fill related data gaps, e.g., by balancing their models using top-down and bottom-up approaches (Müller et al., 2014). On the other hand, the use of dynamic material flow models can provide plausible scenarios of anticipated emissions of TCEs in the future, thereby informing the community of biogeochemist on elements that it should be increasingly monitored in the future.

In particular, in the context of the ongoing debate about moving into the Anthropocene (Crutzen, 2006), information about the EU's contribution to human-induced element mass mobilization compared to their natural cycling are significant. Given that recent trends in global materials flows and stocks are on an upward trend (Krausmann et al., 2009, Krausmann et al., 2017) it is likely that human influence will continue to increase for both TCEs as well as for the major metals (e.g., Fe, Cu, Zn, Al) and bulk materials (e.g., construction materials).

In order to enhance the understanding of Europe's resource base, it is therefore important that EU actors interested in various aspects of TCEs, from their environmental processes, (eco)toxicological issues, and resource management/security of supply aspects, increase the dialogue amongst each other. The current COST action provides a platform to more holistically address the issues of sustainable resource management and establish a long-term network. Future research needs relate to better mapping of both anthropogenic and natural stocks and flows in the EU and globally, and increasing this dialogue between various research communities.

## Appendix A Supplementary data

Supplementary data and information for Figs. 5 and 7 are provided in the appendix. Image 2
