# Supplementary material for: Towards better monitoring of technology critical elements in Europe: Coupling of natural and anthropogenic cycles
Source: Sci Total Environ. 2018 Feb 1;613-614:569–78. doi: 10.1016/j.scitotenv.2017.09.117 (PMC5681708; doi:10.1016/j.scitotenv.2017.09.117)
Supplement: Supplementary file 1 — Supplementary data and information for Figs. 5 and 7 are provided in the appendix. [file mmc1.docx]

**Supplementary Data for:**

**Towards Better Monitoring of Technology Critical Elements in Europe: Coupling of Natural and Anthropogenic Cycles**

Philip Nuss^1^* and Gian Andrea Blengini^1,2^

^1^European Commission, Joint Research Centre (JRC), Directorate D - Sustainable Resources, Ispra 21027, Italy

^2^ Politecnico di Torino, Corso Duca degli Abruzzi 24, 10129 Torino, Italy

*Corresponding author email address: [philip@nuss.me](mailto:philip@nuss.me), Personal Website: [www.philip.nuss.me](http://www.philip.nuss.me)

**1. Data used in Figure 5 in the main manuscript.**

The outflows from the use phase of niobium in transportation are modeled using the depletion time model of (Graedel et al., 2012). Data collection and assumption are described in (Nuss et al., 2014) and include historical global mine production data, end use shares over time, end-of-life recycling rates, product lifetimes (10 years for automobiles) and life-time distributions (a normal distribution with standard deviation of 30% is used), and data on losses to tailings and slags. Details are given in section 2.4.5 of the supporting information of (Nuss et al., 2014). Data of calculated total inflows and outflows of niobium in transportation are shown in Table S1.

**Table S1.** Data of global niobium inflows into use and flows out of use (used in Figure 5 of the main manuscript). [kg/yr] (in niobium content).

| **Year** | **Flow into use** | **Outflow** | **Year** | **Flow into use** | **Outflow** |
| --- | --- | --- | --- | --- | --- |
| 1964 | 248,000 | 571 | 1990 | 3,174,544 | 1,769,420 |
| 1965 | 312,303 | 1,688 | 1991 | 3,371,292 | 1,881,977 |
| 1966 | 506,895 | 4,600 | 1992 | 3,445,448 | 2,001,663 |
| 1967 | 517,438 | 10,483 | 1993 | 3,106,881 | 2,131,429 |
| 1968 | 500,556 | 21,685 | 1994 | 3,798,657 | 2,274,355 |
| 1969 | 672,493 | 41,416 | 1995 | 3,935,408 | 2,427,452 |
| 1970 | 867,951 | 72,616 | 1996 | 4,202,549 | 2,588,167 |
| 1971 | 412,486 | 116,111 | 1997 | 5,164,228 | 2,754,295 |
| 1972 | 656,539 | 173,382 | 1998 | 6,437,776 | 2,924,591 |
| 1973 | 1,561,893 | 243,147 | 1999 | 6,347,033 | 3,098,651 |
| 1974 | 1,062,868 | 319,086 | 2000 | 6,602,285 | 3,284,651 |
| 1975 | 955,115 | 398,463 | 2001 | 8,116,365 | 3,496,922 |
| 1976 | 1,158,186 | 478,265 | 2002 | 8,846,368 | 3,749,928 |
| 1977 | 1,133,480 | 556,702 | 2003 | 10,673,462 | 4,063,141 |
| 1978 | 1,262,052 | 634,810 | 2004 | 8,225,465 | 4,440,210 |
| 1979 | 1,776,449 | 714,704 | 2005 | 12,050,811 | 4,898,466 |
| 1980 | 1,888,793 | 796,595 | 2006 | 14,740,187 | 5,430,877 |
| 1981 | 1,976,195 | 880,606 | 2007 | 17,495,365 | 6,035,718 |
| 1982 | 1,632,721 | 965,137 | 2008 | 28,638,931 | 6,733,615 |
| 1983 | 1,498,223 | 1,050,444 | 2009 | 19,672,816 | 7,503,137 |
| 1984 | 2,224,735 | 1,139,296 | 2010 | 15,760,662 | 8,391,411 |
| 1985 | 2,453,827 | 1,233,074 | 2011 | 16,495,448 | 9,434,752 |
| 1986 | 2,551,529 | 1,333,767 | 2012 | 20,048,419 | 10,661,192 |
| 1987 | 1,970,496 | 1,439,302 | 2013 | 19,354,432 | 12,056,263 |
| 1988 | 3,128,830 | 1,549,981 | 2014 | 22,853,819 | 13,570,594 |
| 1989 | 2,865,990 | 1,659,517 | 2015 | 22,624,415 | 15,081,588 |

**2. Data used in Figure 7 in the main manuscript.**

All data underlying Figure 7 in the main manuscript are provided in Tables S2 and S3 of this supplementary information.

**Table S2.** Element flows in the EU-28 in 2012/2013 from the EU Raw Materials System Analysis Study (BIO by Deloitte, 2015) [kg/yr].

| **Material Name** | **Imports** | | | | | **Exports** | | | | | **Domestic Extraction** | **Recycling** |
| --- | --- | --- | --- | --- | --- | --- | --- | --- | --- | --- | --- | --- |
| **Details** | **[A] Primary material** | **[B] Secondary material** | **[C] Processed material** | **[D] Product** | **[E] Waste** | **[F] Primary material** | **[G] Secondary material** | **[H] Processed material** | **[I] Product (including for reuse)** | **[J] Waste** | **[K]** | **[L]** |
| **Pd** | 1.50E+04 | 2.72E+04 | 7.08E+04 | 1.53E+04 | 0.00E+00 | 2.50E+02 | 0.00E+00 | 5.14E+04 | 3.45E+04 | 4.49E+03 | 7.87E+02 | 1.09E+04 |
| **Pt** | 1.50E+04 | 1.75E+04 | 7.23E+04 | 1.03E+04 | 0.00E+00 | 2.50E+02 | 0.00E+00 | 4.48E+04 | 3.60E+04 | 8.24E+03 | 8.59E+02 | 1.36E+04 |
| **Sb** | 4.56E+05 | 1.75E+04 | 2.46E+07 | 1.18E+07 | 9.88E+05 | 0.00E+00 | 0.00E+00 | 6.54E+05 | 2.11E+07 | 2.01E+06 | 0.00E+00 | 9.71E+06 |
| **Cr** | 8.02E+08 | 9.01E+07 | 2.78E+08 | 1.11E+08 | 8.96E+07 | 3.23E+06 | 8.44E+07 | 4.63E+08 | 2.21E+08 | 8.59E+06 | 3.77E+08 | 5.17E+08 |
| **W** | 2.58E+06 | 7.26E+04 | 1.09E+07 | 8.11E+06 | 0.00E+00 | 1.18E+06 | 0.00E+00 | 1.03E+05 | 9.85E+06 | 0.00E+00 | 2.73E+06 | 1.11E+07 |
| **Rh** | 2.00E+03 | 8.76E+03 | 5.29E+03 | 8.23E+02 | 0.00E+00 | 5.00E+01 | 0.00E+00 | 9.66E+03 | 4.05E+03 | 9.48E+02 | 6.06E+01 | 1.68E+03 |
| **In** | 1.72E+04 | 8.31E+03 | 6.13E+04 | 8.14E+04 | 2.91E+03 | 2.67E+03 | 0.00E+00 | 1.25E+04 | 5.20E+04 | 1.45E+04 | 1.13E+05 | 3.69E+03 |
| **Li** | 7.18E+06 | 0.00E+00 | 1.42E+07 | 8.43E+06 | 0.00E+00 | 0.00E+00 | 4.15E+04 | 0.00E+00 | 5.18E+06 | 7.23E+05 | 2.20E+06 | 1.60E+04 |
| **Nb** | 1.60E+05 | 4.53E+05 | 1.44E+07 | 1.39E+06 | 5.43E+02 | 0.00E+00 | 0.00E+00 | 9.46E+05 | 4.80E+06 | 2.96E+05 | 0.00E+00 | 1.87E+06 |
| **B** | 1.57E+07 | 0.00E+00 | 6.01E+07 | 1.16E+07 | 7.56E+04 | 0.00E+00 | 4.72E+02 | 0.00E+00 | 1.35E+07 | 3.62E+04 | 0.00E+00 | 4.65E+05 |
| **Co** | 1.02E+07 | 0.00E+00 | 5.61E+05 | 1.14E+07 | 1.51E+04 | 1.11E+05 | 0.00E+00 | 4.76E+06 | 4.79E+06 | 1.02E+06 | 1.53E+06 | 6.32E+06 |
| **P** | 5.11E+08 | 0.00E+00 | 3.06E+08 | 1.00E+09 | 1.35E+05 | 0.00E+00 | 1.79E+05 | 3.71E+07 | 2.24E+08 | 3.77E+05 | 8.71E+07 | 1.80E+08 |
| **Tb** | 1.37E+04 | 0.00E+00 | 6.34E+04 | 3.25E+04 | 1.26E+03 | 0.00E+00 | 0.00E+00 | 2.22E+04 | 2.68E+04 | 2.52E+02 | 0.00E+00 | 2.17E+04 |
| **Eu** | 9.19E+03 | 0.00E+00 | 4.59E+04 | 3.27E+04 | 1.69E+03 | 0.00E+00 | 0.00E+00 | 2.92E+04 | 2.17E+04 | 0.00E+00 | 0.00E+00 | 3.35E+04 |
| **Dy** | 4.02E+04 | 0.00E+00 | 1.69E+05 | 5.67E+04 | 7.98E+02 | 0.00E+00 | 0.00E+00 | 1.72E+04 | 6.36E+04 | 1.27E+03 | 0.00E+00 | 0.00E+00 |
| **Be** | 0.00E+00 | 0.00E+00 | 5.08E+04 | 1.23E+05 | 1.51E+03 | 0.00E+00 | 0.00E+00 | 0.00E+00 | 2.19E+04 | 2.46E+04 | 0.00E+00 | 0.00E+00 |
| **Y** | 1.02E+05 | 0.00E+00 | 6.87E+05 | 4.46E+05 | 1.85E+04 | 0.00E+00 | 0.00E+00 | 3.13E+05 | 3.15E+05 | 6.77E+02 | 0.00E+00 | 3.62E+05 |
| **Ga** | 7.74E+05 | 5.23E+03 | 4.15E+04 | 4.23E+04 | 4.27E+02 | 0.00E+00 | 0.00E+00 | 6.38E+04 | 2.74E+04 | 2.04E+02 | 9.42E+04 | 2.68E+04 |
| **Nd** | 1.78E+05 | 0.00E+00 | 8.47E+05 | 3.85E+05 | 1.08E+04 | 0.00E+00 | 0.00E+00 | 8.27E+04 | 3.32E+05 | 7.83E+03 | 0.00E+00 | 1.39E+04 |
| **Ge** | 5.05E+04 | 3.27E+02 | 1.75E+04 | 1.06E+04 | 1.58E+02 | 0.00E+00 | 0.00E+00 | 1.03E+04 | 2.11E+04 | 9.23E+03 | 0.00E+00 | 1.58E+04 |
| **Er** | 0.00E+00 | 0.00E+00 | 3.63E+04 | 3.84E+03 | 0.00E+00 | 0.00E+00 | 0.00E+00 | 0.00E+00 | 4.85E+03 | 0.00E+00 | 0.00E+00 | 0.00E+00 |
| **Mg** | 1.11E+08 | 0.00E+00 | 1.93E+07 | 3.78E+07 | 5.05E+04 | 0.00E+00 | 1.20E+04 | 1.79E+06 | 3.84E+07 | 3.04E+04 | 0.00E+00 | 2.16E+07 |
| **Si** | 6.53E+07 | 0.00E+00 | 4.45E+08 | 8.58E+07 | 0.00E+00 | 1.15E+08 | 0.00E+00 | 4.92E+07 | 1.61E+08 | 1.84E+05 | 3.11E+08 | 0.00E+00 |

**Table S3.** Calculation of the ratio of EU-28 anthropogenic mobilization fluxes (see Figure 7 in the main manuscript) [Gg/yr].

| **Flow Name** | **DMI (incl. secondary material inputs)**  (BIO by Deloitte, 2015) | **DMI (excl. secondary material inputs)**  (BIO by Deloitte, 2015) | **DMC (excl. functional recycling)**  (BIO by Deloitte, 2015) | **DMC (incl. functional recycling)**  (BIO by Deloitte, 2015) | **Global Natural flow**  (Sen and Peucker-Ehrenbrink, 2012) | **Log [DMI (incl. secondary material inputs) (EU-28) / Natural Flux (Global)]** | **Log [DMI (excl. secondary material inputs) (EU-28) / Natural Flux (Global)]** | **Log [DMC (excl. functional recycling) (EU-28) / Natural Flux (Global)]** | **Log [DMC (incl. functional recycling) (EU-28) / Natural Flux (Global)]** |
| --- | --- | --- | --- | --- | --- | --- | --- | --- | --- |
| **Details** | Flows from Table S2: [A]+[B]+[C]+[D]+[E]+[K]+[L] | Flows from Table S2: [A]+[C]+[D]+[K] | Flows from Table S2: Imports + Domestic Extraction - Exports | Flows from Table S2: Imports + Domestic Extraction + Functional Recycling - Exports | Flows from Table S1 in (Sen and Peucker-Ehrenbrink, 2012) |  | **Shown in Figure 7 of the main manuscript** | **Shown in Figure 7 of the main manuscript** | - |
| **Pd** | 0.14 | 0.10 | 0.038 | 0.049 | 0.06 | 0.37 | **0.23** | **-0.19** | -0.08 |
| **Pt** | 0.13 | 0.10 | 0.027 | 0.040 | 0.06 | 0.33 | **0.22** | **-0.35** | -0.17 |
| **Sb** | 48 | 37 | 14 | 24 | 34 | 0.15 | **0.04** | **-0.38** | -0.16 |
| **Cr** | 2,264 | 1,568 | 967 | 1,483 | 8,129 | -0.56 | **-0.71** | **-0.92** | -0.74 |
| **W** | 36 | 24 | 13 | 24 | 203 | -0.76 | **-0.92** | **-1.19** | -0.92 |
| **Rh** | 0.02 | 0.008 | 0.0022 | 0.0039 | 0.07 | -0.58 | **-0.93** | **-1.49** | -1.25 |
| **In** | 0.29 | 0.27 | 0.20 | 0.21 | 6 | -1.32 | **-1.34** | **-1.47** | -1.46 |
| **Li** | 32 | 32 | 26 | 26 | 2,083 | -1.81 | **-1.81** | **-1.90** | -1.90 |
| **Nb** | 18 | 16 | 10 | 12 | 1,213 | -1.82 | **-1.88** | **-2.07** | -2.00 |
| **B** | 88 | 87 | 74 | 74 | 15,932 | -2.26 | **-2.26** | **-2.33** | -2.33 |
| **Co** | 30 | 24 | 13 | 19 | 5,617 | -2.27 | **-2.38** | **-2.64** | -2.46 |
| **P** | 2,087 | 1,907 | 1,646 | 1,826 | 605,625 | -2.46 | **-2.50** | **-2.57** | -2.52 |
| **Tb** | 0.13 | 0.11 | 0.062 | 0.083 | 65 | -2.69 | **-2.77** | **-3.02** | -2.89 |
| **Eu** | 0.12 | 0.088 | 0.039 | 0.072 | 89 | -2.86 | **-3.01** | **-3.36** | -3.09 |
| **Dy** | 0.27 | 0.27 | 0.18 | 0.18 | 354 | -3.12 | **-3.12** | **-3.28** | -3.28 |
| **Be** | 0.18 | 0.17 | 0.13 | 0.13 | 304 | -3.24 | **-3.24** | **-3.37** | -3.37 |
| **Y** | 1.6 | 1.2 | 0.62 | 0.99 | 2,223 | -3.14 | **-3.26** | **-3.55** | -3.35 |
| **Ga** | 0.98 | 0.95 | 0.87 | 0.89 | 1,718 | -3.24 | **-3.26** | **-3.30** | -3.28 |
| **Nd** | 1.4 | 1.4 | 1.0 | 1.0 | 2,627 | -3.26 | **-3.27** | **-3.42** | -3.41 |
| **Ge** | 0.09 | 0.079 | 0.039 | 0.054 | 162 | -3.23 | **-3.31** | **-3.62** | -3.47 |
| **Er** | 0.04 | 0.04 | 0.035 | 0.035 | 233 | -3.76 | **-3.76** | **-3.82** | -3.82 |
| **Mg** | 190 | 168 | 128 | 150 | 3,046,261 | -4.21 | **-4.26** | **-4.38** | -4.31 |
| **Si** | 907 | 907 | 582 | 582 | 29,337,714 | -4.51 | **-4.51** | **-4.70** | -4.70 |
| **Explanation** | Anthropogenic element flows due to both primary and secondary material inputs in the EU-28. | Anthropogenic element flows only due to primary material inputs in the EU-28. | Anthropogenic element flows due to EU consumption excluding secondary materials from functional recycling within EU-28 borders. | Anthropogenic element flows due to EU consumption including secondary materials from functional recycling within EU-28 borders. | Global natural element flows consisting of riverine flux to ocean, Eolian dust, seaspray, net primary production (NPP), extraterrestrial matter, volcano, and soil erosion. | - | **-** | **-** | - |

**3. References**

BIO by Deloitte, 2015. Study on Data for a Raw Material System Analysis: Roadmap and Test of the Fully Operational MSA for Raw Materials. Prepared for the European Commission, DG GROW.

Graedel, T.E., Barr, R., Chandler, C., Chase, T., Choi, J., Christoffersen, L., Friedlander, E., Henly, C., Jun, C., Nassar, N.T., Schechner, D., Warren, S., Yang, M., Zhu, C., 2012. Methodology of Metal Criticality Determination. Environ. Sci. Technol. 46, 1063–1070. doi:10.1021/es203534z

Nuss, P., Harper, E.M., Nassar, N.T., Reck, B.K., Graedel, T.E., 2014. Criticality of Iron and Its Principal Alloying Elements. Environ. Sci. Technol. 48, 4171–4177. doi:10.1021/es405044w

Sen, I.S., Peucker-Ehrenbrink, B., 2012. Anthropogenic Disturbance of Element Cycles at the Earth’s Surface. Environ. Sci. Technol. 46, 8601–8609. doi:10.1021/es301261x
